# Validating Age-Related Functional Imaging Changes in Verbal Working Memory with Acute Stroke

**DOI:** 10.3233/BEN-2011-0331

**Published:** 2011-08-29

**Authors:** Timothy B. Meier, Lin Naing, Lisa E. Thomas, Veena A. Nair, Argye E. Hillis, Vivek Prabhakaran

**Affiliations:** ^1^Neuroscience Training ProgramUniversity of Wisconsin-MadisonMadisonWIUSA; ^2^School of Medicine and Public HealthUniversity of Wisconsin-MadisonMadisonWIUSA; ^3^Emergency MedicineMassachusetts General Hospital and Brigham and Women's HospitalMAUSA; ^4^Department of RadiologyUniversity of Wisconsin-MadisonMadisonWIUSA; ^5^Department of NeurologyJohns Hopkins University School of MedicineBaltimoreMAUSA

**Keywords:** Working memory, aging, lesion, stroke

## Abstract

Functional imaging studies consistently find that older adults recruit bilateral brain regions in cognitive tasks that are strongly lateralized in younger adults, a characterization known as the Hemispheric Asymmetry Reduction in Older Adults model. While functional imaging displays what brain areas are active during tasks, it cannot demonstrate what brain regions are necessary for task performance. We used behavioral data from acute stroke patients to test the hypothesis that older adults need both hemispheres for a verbal working memory task that is predominantly left-lateralized in younger adults. Right-handed younger (age ≥ 50, *n* = 7) and older adults (age > 50, *n* = 21) with acute unilateral stroke, as well as younger (*n* = 6) and older (*n* = 13) transient ischemic attack (TIA) patients, performed a self-paced verbal item-recognition task. Older patients with stroke to either hemisphere had a higher frequency of deficits in the verbal working memory task compared to older TIA patients. Additionally, the deficits in older stroke patients were mainly in retrieval time while the deficits in younger stroke patients were mainly in accuracy. These data suggest that bihemispheric activity is necessary for older adults to successfully perform a verbal working memory task.

## 1. Introduction

Functional imaging is a valuable tool in measuring brain activity during cognitive processes in humans. As such, there is a vast body of literature using techniques such as fMRI to investigate working memory [12,13, 50,56]. While imaging data provides information on what areas of the brain are activated during different cognitive processes, it cannot provide information on what areas of the brain are essential for the process being tested. It is important to validate results obtained from functional imaging studies, and one way to do so in humans is with lesion studies [31,41]. Lesion effects can be studied in stroke patients. Chronic stroke patients have been studied extensively to test the necessity of the affected area for many cognitive tasks, including working memory [for review, see [31]]. Deficits in cognitive performance can be measured and linked to the stroke-altered areas. While studies of chronic stroke patients can provide valuable information, one drawback is that considerable reorganization is known to occur over time [38,53]. The use of acute stroke patients provides the unique opportunity to test the effect of the lesion on performance before substantial reorganization can occur. Diffusion-weighted imaging (DWI) and perfusion weighted imaging (PWI) can be used to

ISSN 0953-4180/11/$27.50 © 2011 – IOS Press and the authors. All rights reserved determine structural damage and blood flow abnormalities, respectively, allowing for identification of areas affected by acute stroke thereby directly linking the affected area to any observed deficit in performance [2, 23]. In this study we used acute stroke patients to validate an aging model of cognition.

Normal aging leads to changes in brain anatomy and physiology that are reflected in changes in cognition [16]. These changes have been documented in numerous functional imaging studies [for review, see [15]]. One example of aging-related changes in the prefrontal cortex, as documented by several functional imaging studies, is a decrease in asymmetry of brain activation in older adults for cognitive tasks that are known to be predominantly unilateral in young adults [5,6,19,39]. This consistently observed phenomenon has been characterized as the Hemispheric Asymmetry Reduction in Older Adults, or HAROLD model [7]. The HAROLD model has been supported by studies of several different cognitive facilities including episodic memory, working memory, perception, and inhibitory control [for review, see [7]]. Although HAROLD was initially proposed for the prefrontal cortex, there is evidence the model applies to more posterior regions as well. For example, in temporal and parietal regions, age-dependent reductions in activation asymmetries have been observed during a visual facial-affect perception task [22], face memory and processing tasks [20,21], in inhibitory control tasks [30, 32], in language processing tasks [3], and in a verbal working memory task [40]. In addition, age-related reductions in activation asymmetries have also been observed in the occipital lobe during an attention task [27] and in the hippocampus during autobiographical memory task [28]. Furthermore, age-related asymmetry in fronto-parietal connections has also been demonstrated. In a study by Li et al., prefrontal cortex was shown to have strong functional connectivity to the ipsilateral parietal area during a working memory task, as well as during rest, and that this connectivity was reduced in older participants [26]. Also, Charlton et al. found that most of the age-related variance in working memory is explained by the integrity of the fronto-parietal white matter tracts [10]. Together, these studies suggest that the HAROLD model likely extends to more posterior regions in addition to the PFC. Although age-related increased activation has been demonstrated across the cortex for several different tasks, there is little evidence that this increase in activation represents areas essential for task performance.

In this study, we use data collected from acute ischemic stroke patients to validate neuroimaging findings that suggest that older adults need both hemispheres to successfully complete a verbal working memory task. The characterization of activations observed in young and old subjects known as the HAROLD model predicts that older subjects need both hemispheres for tasks that are considered lateralized in young subjects [7]. In young normal adults, functional imaging studies have shown that the left hemisphere is responsible for verbal working memory [24,33,35, 49] while the right hemisphere has been shown to be involved in verbal working memory only during high demand tasks [11,44]. Studies have found that the lateralization of verbal WM extends into posterior regions of the brain as well. In verbal delayed match to sample tasks, Smith et al. observed left lateralization in PFC, supplementary motor area, premotor cortex, and parietal cortex [51]. In a similar verbal WM task, Salmon et al. observed left lateralized activity in the PFC and parietal cortex [47].

Consistent with the HAROLD model, Reuter-Lorenz et al. [39] found that older adults displayed bilateral PFC activity for verbal working memory while young adults showed more lateralized activity. In a subsequent study, Reuter-Lorenz observed bilateral activation in older adults in the parietal cortex during a verbal working WM task while younger adults had activation in the left parietal cortex [40]. The bilateral activation observed in older adults suggests that both hemispheres are necessary for successful performance during verbal working memory tasks. To our knowledge, the HAROLD model has not been validated with lesion data, although it has been investigated in regards to episodic memory using transcranial magnetic imaging [29,42]. Here we test the hypothesis that older individuals need this bilateral activation to perform successfully in a cognitive task that is known to be lateralized in normal young adults by comparing the effect of left or right hemisphere stroke on old patients. If both hemispheres are needed in old adults for successful verbal working memory task performance, stroke localized to either side should negatively affect task performance.

## 2. Methods

Participants for this study were recruited in order to investigate the lateralization of working memory processes in young and old adults. An extension of the analyses were carried out from the original published data and results which were based on findings regarding

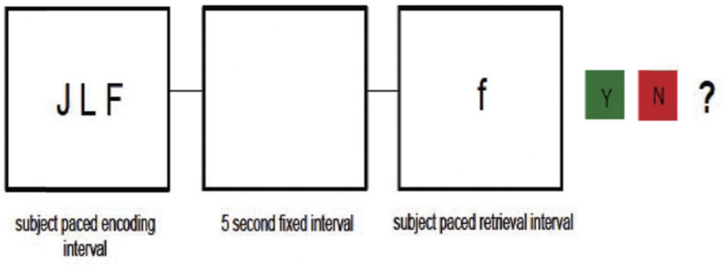

Fig. 1. Participants performed a self-paced verbal working memory tasks. Subjects were asked to encode a set of three (low load), four, five, or six (high load) letters upper-case letters. After a 5 second delay, subjects were asked to decide whether a single lower-case letter was included in the encoding period.

the lateralization of spatial and verbal working memory regardless of age, reported previously by Philipose and colleagues [36]. Consenting right-handed adults with acute ischemic stroke were studied within three days of symptom-onset and within 24 hours of hospitalization. Only acute stroke patients were included in order to limit post-stroke reorganization. In addition, consenting right-handed adults with transient ischemic attack (TIA; defined as resolution of symptoms within 24 hours and exclusion of stroke by MRI) were enrolled. These patients provided a unique control population for acute stroke patients, as they shared similar hospitalization stressors, age, and vascular risk factors with the stroke patients. Any differences in performance between stroke patients and TIA patients can be attributed to the actual lesion. Patients with the following conditions were excluded: (1) premorbid dementia, neurological, or psychiatric disease; (2) contraindication for MRI; (3) diminished level of consciousness; (4) lack of premorbid proficiency in English; (5) visual field deficits as determined by medical records and bedside examination; (6) motor deficits precluding performance of computer tasks; (7) language deficits that interfered with task comprehension; or (8) left neglect, defined by $>10\%$ errors on the left half of the page in line cancellation. The following MRI sequence were used on all patients as part of the Johns Hopkins Acute Stroke Protocol: diffusion weighted imaging (DWI) and perfusion weighted imaging (PWI), axial T2, fluid attenuated inversion recovery (FLAIR), gradient echo (GE), and apparent diffusion coefficient (ADC) maps. The reported analyses used DWI (after confirming the acuity of the lesion on ADC maps) and PWI (co-registered to T2 to provide anatomical boundaries that are less visible on PWI). Whole brain coverage was obtained by DWI and PWI scans of 5 mm thick slices. Volumes of tissue dysfunction on DWI and/or PWI were calculated using the Image J program. If a patient had imaging abnormalities on both DWI and PWI, the larger calculated volume was used. Volume of PWI abnormality was measured on time to peak maps, defining hypoperfused tissue as voxels where there was >4 seconds delay in time to peak arrival of contrast, relative to the homologous region in the normal hemisphere.

Stroke was defined as acute infarct on DWI and/or hypoperfusion on PWI with corresponding neurological deficits, while TIA was defined as normal DWI/PWI and resolution of neurological deficits within 24 hours. In the current study, stroke and TIA patients were classified by age as either young or old. The old group included all patients of age $> 50$ years, while the young group included all individuals $\leqslant 50$ years of age. The age cut-off of 50 years was chosen here based on the finding that white matter volume is known to start declining during the fifth decade of life [17,18]. Older stroke patients were further separated as either left hemisphere or right hemisphere depending on the location of the stroke.

Behavioral tasks performed were described in detail elsewhere [36]. Briefly, patients performed a self-paced computerized verbal working memory task [52]. Subjects were asked to remember 3, 4, 5, or 6 upper-case letters and presented with a lower-case probe letter after the 5 second delay (Fig. 1). Responses were made with their dominant hand, unless they preferred to use the opposite hand due to right hand or arm weakness. Responses that were two standard deviations from the mean of a participant's total trials were excluded from the analysis. This removed trials that were interrupted by other factors related to the bed-side testing.

To contrast performance in the WM tasks, preplanned comparisons of deficits in encoding time, response time, and accuracy (percent correct) were carried out between groups across all loads (that is, the 3, 4, 5, and 6 letter conditions combined). Given the known effect of WM load on performance [44], pre-

Table 1
Group Demographics: Listed are the ages and number of patients in the analysis. Educational information was only available for eight stroke patients (four old left and four old right), two young stroke patients, seven old TIA patients, and three young stroke patients. Posterior refers to temporal, parietal, and/or occipital cortex. Information regarding stroke volume was not available for one old left stroke patient, two old right stroke patients, and one young stroke patient. The old right stroke group had a larger average lesion volume than the old left stroke group however this was neither significant nor trending towards significance. The discrepancy was due in large part to one old right stroke patient with lesion volume of 155,796.2 mm$^3$. The remaining old right stroke patients had a average lesion volume of 16,597.3 mm$^3$

| Patient group | Older stroke | Younger stroke | Old left stroke | Old right stroke | Older TIA | Younger TIA |
|---|---|---|---|---|---|---|
| Number | 18 | 6 | 8 | 10 | 13 | 6 |
| % Male | 78% | 50% | 87.5% | 70% | 46% | 33% |
| Age range | 53–79 | 38–50 | 53–64 | 53–79 | 52–84 | 21–50 |
| Age mean and SEM | $61.9 \pm 1.6$ | $43 \pm 1.67$ | $59.8 \pm 1.5$ | $63.6 \pm 2.6$ | $64.0 \pm 3.1$ | $41.5 \pm 4.3$ |
| Years of education | $19.88 \pm 1.125$ | $21 \pm 0$ | $21 \pm 0$ | $18.75 \pm 2.25$ | $15.29 \pm 2.09$ | $15 \pm 3$ |
| Posterior stroke(n) | 9 | 0 | 5 | 4 | – | – |
| Frontal stroke (n) | 3 | 0 | 1 | 2 | – | – |
| Frontal and posterior stroke (n) | 3 | 3 | 1 | 2 | – | – |
| Sub-cortical stroke (n) | 3 | 3 | 1 | 2 | – | – |
| Stroke Volume mm$^3$ (mean, SEM) | $22502 \pm 10724$ | $25519 \pm 10610$ | $9365 \pm 3632$ | $33997 \pm 19518$ | – | – |

planned comparisons were also done at the low (3 letters) and high (6 letters) loads alone. A composite WM score was calculated by summing the deviations from the mean, as measured by z-scores, for encoding time, response time, and accuracy. Deficits were defined as having a score more than two standard deviations from the mean scores taken from 20 healthy normal volunteers recruited for a different study that were given the same behavioral protocol as patients. Chi-squared tests were performed as different groups (e.g. left stroke, tia) were compared with respect to presence or absence of behavioral deficits in which frequencies of deficits were compared across groups using contingency tables. Contingency tables were used due to the nonparametric nature of the data in order to reduce the effect of the large amount of between subject variability in the performance measures. Due to the low n for some comparisons, Fisher's Exact Tests (FET) were used when chi-square tests were not appropriate (i.e. when the expected values were $< 5$).

## 3. Results

Data for this study were reported in a previous study by Philipose et al. [36]. The purpose of the previous study was to investigate lateralization of working memory processes regardless of age. From those data, 28 stroke patients and 19 TIA patients were used for the retrospective analyses reported here. Age ranged from 38–79 years ($57.17 \pm 2.1$; mean and SEM) for stroke patients and 21–84 years ($56.89 \pm 3.5$) for TIA patients. Information regarding gender, lesion volume, lesion location, and education level is included in Table 1. A two-sample t-test comparing the mean lesion volume between older and younger stroke patients was not significant, nor was the test between older left stroke patients and older right stroke patients ($t(18) = -0.15$, $p = 0.84$, $t(13) = 1.24$, $p = 0.25$). Thus, infarct volume was roughly equivalent between groups. A two-sample t-test comparing education levels between groups revealed that older stroke patients had a trend of having more years of education compared to older TIA patients ($t(13) = 2.0$, $p = 0.066$). Education levels were not statistically different between young stroke patients and young TIA patients.

### 3.1. *Age effects*

To examine effects of age on performance, stroke and TIA patients were split into young (up to 50 years old) and old (51 years and older) groups. Demographics are presented in Table 1.

#### 3.1.1. *Across all loads*

To determine whether there was an age-dependent effect of stroke on verbal WM collapsed across all loads, relative performance of both the young and old stroke groups was compared to age-matched TIA patients (Fig. 2). While both the old stroke group and the young stroke group displayed a trend of having a higher frequency of deficits than age-matched TIA patients on the composite WM measure ($p = 0.051$and $p = 0.061$, FET, respectively), the deficits seemed to be driven by different aspects of the task. For younger stroke patients, there was a significantly higher frequency of deficits in accuracy compared to young TIA patients ($p < 0.05$, FET). In contrast, older stroke patients displayed more deficits in response time than old TIA patients ($\chi^2 = 6.482$, $df = 1$, $p < 0.05$).

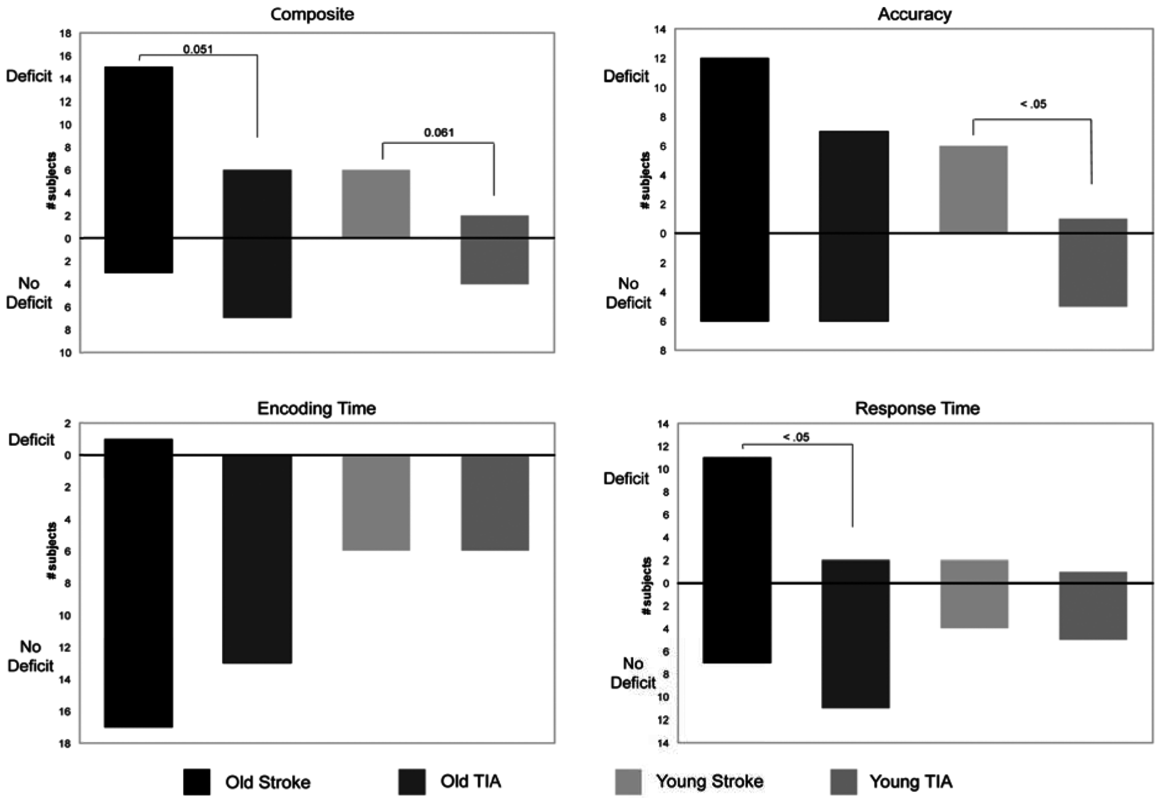

Fig. 2. Number of old and young patients with stroke to either hemisphere with and without deficits in the composite score, accuracy, encoding time, and response time. Old and young strokes involving the left or right hemisphere displayed a trend of having more deficits in composite score than age-matched TIA patients. The overall impairment in old stroke patients was driven by deficits in response time with respect to old TIA patients. In contrast, overall impairment in young stroke patients was driven by deficits in accuracy with respect to young TIA patients.

#### *3.1.2. Low and high verbal loads*

In order to investigate the specific contribution of load on these effects, performance on the WM task was also compared at the high load and low load conditions. At the low verbal load (3 letters; Fig. 3a), older stroke patients had significantly more performance deficits than older TIA patients in encoding time and response time ($\chi^2 = 5.134$, $df = 1, p < 0.05$; $\chi^2 = 8.016$, $df = 1$, $p < 0.005$, respectively). In contrast, young stroke patients did not differ in terms of performance compared to young TIA patients at the low load. As would be expected, deficits were observed for each group at the high verbal load (Fig. 3b). Old stroke patients had significantly more deficits than old TIA patients in the composite score and in response time ($p < 0.05$, FET and $\chi^2 = 8.016$, $df = 1$, $p < 0.005$, respectively). While the deficits in older stroke patients were driven heavily by deficits in response time, younger stroke patients displayed a trend of having more deficits than young TIA patients in both accuracy and response time ($p = 0.061$ and $p = 0.08$, FET, respectively).

### *3.2. Age and hemisphere effects*

In order to test the hypothesis that older subjects need both hemispheres in order to perform a WM task that younger subjects use predominantly one hemisphere for, the performance of old left and old right stroke patients was compared to the performance of old TIA patients. For this analysis all strokes were classified as either left or right hemisphere. Demographics are presented in Table 1.

#### *3.2.1. Across all loads*

The performance of each group on the verbal WM task across all loads is shown in Fig. 4. Old patients in the right stroke group displayed a trend of having a higher frequency deficits in the composite score compared to old TIA patients ($p = 0.074$, FET). Both old left and right stroke patients had a higher frequency of deficits in response time than old TIA patients, with the old right comparison being significant ($p < 0.05$, FET) and the old left comparison trending towards significance ($p = 0.055$).

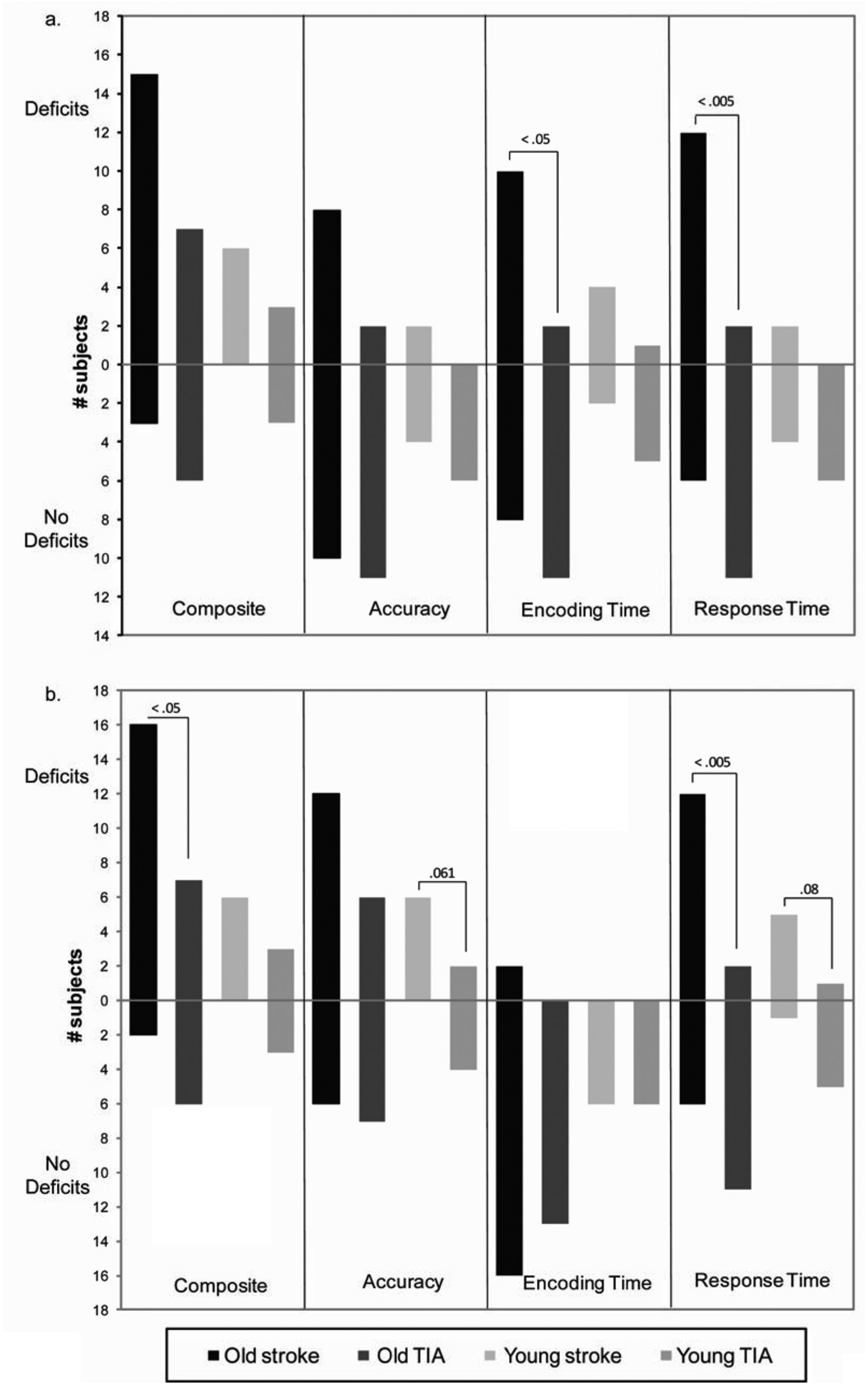

Fig. 3. Number of old and young patients with stroke to either hemisphere with and without deficits at the low verbal load and the high verbal load. At the low load ($a$), old stroke patients were significantly more impaired in encoding time and response time than old TIA patients, while young stroke patients did not differ from young TIA patients in terms of performance. At the high load ($b$), old stroke patients were significantly more impaired in the composite score and in response time with respect to old TIA patients. Young stroke patients displayed a trend of more deficits in both accuracy and in response time than young TIA patients.

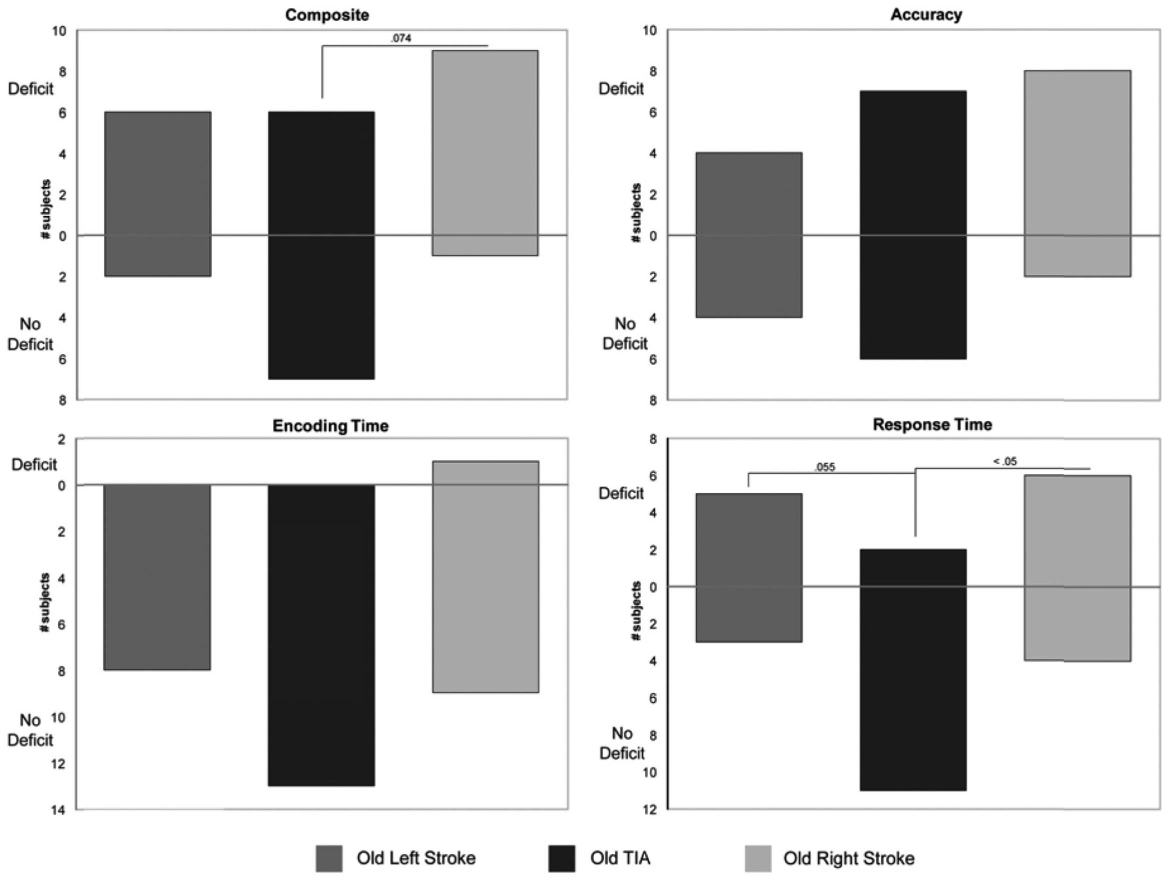

Fig. 4. Number of old patients with left and right unilateral stroke with and without deficits across all verbal loads. Old right stroke patients had a trend of more deficits in the composite score and significantly more deficits in response time than old TIA patients. Old left stroke patients had a trend of more deficits in response time than old TIA patients.

*3.2.2. Low and high verbal loads*

In order to compare performance between the old stroke groups and control group performance on the verbal WM task was compared at the low and high WM loads alone. A similar pattern emerged at the low and high verbal load compared to that observed across all loads. At the low verbal load (Fig. 5a) old right stroke patients displayed a trend towards having a higher frequency of deficits in the composite score than old TIA patients ($p = 0.089$, FET). In addition, old right stroke patients had significantly more deficits than old TIA patients in encoding time ($p < 0.05$, FET) and response time ($p < 0.05$, FET). Although no effects on the composite score were observed for old left stroke patients, they did display a trend towards significance for having more deficits than old TIA patients in response time ($p = 0.056$, FET).

At the high verbal load, older right stroke patients had significantly more deficits in response time than older TIA patients ($p < 0.005$, FET) and also displayed a trend towards more deficits as measured by the composite score ($p = 0.089$, FET, Fig. 5b). Old left stroke patients did not display any statistical difference in the frequency of deficits in any measure compared to old TIA patients.

## 4. Discussion

In the present study, we used acute unilateral ischemic stroke patients to validate a characterization of fMRI activation patterns observed in older adults. In healthy young adults, verbal working memory is associated predominantly with the left hemisphere, with the right hemisphere only being involved at higher loads [11,33,37,43]. The HAROLD model posits that PFC activity for known lateralized cognitive tasks, such as working memory, becomes more bilateral in older

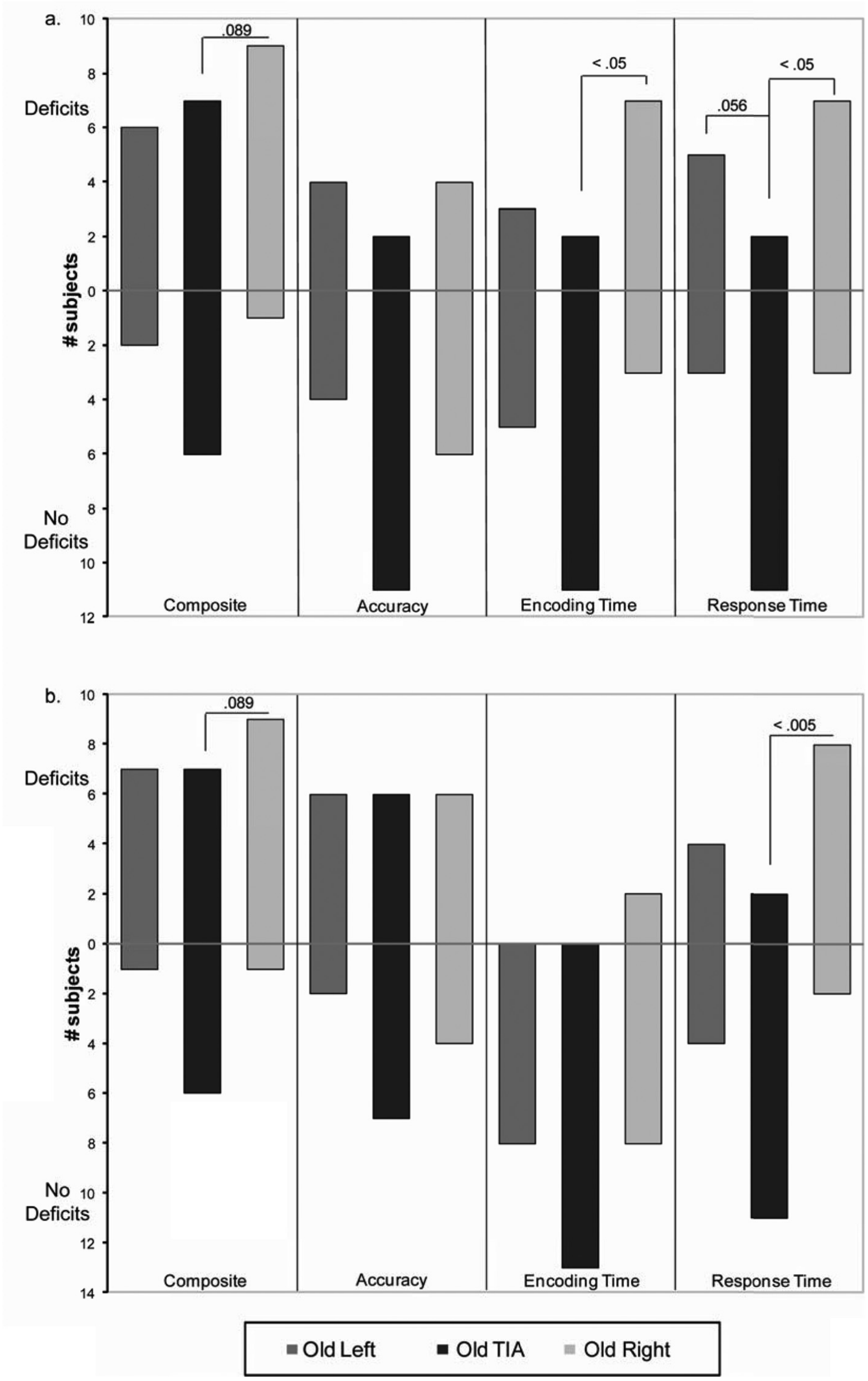

Fig. 5. Number of old patients with left and right unilateral stroke with and without deficits at the low and high verbal load. At the low load ($a$), old right stroke patients displayed a trend towards more deficits in the composite score, and had significantly more deficits in encoding time and response time than old TIA patients. Old left stroke patients had a trend towards more deficits than old TIA patients in response time. At the high load ($b$), old right stroke patients displayed a trend towards more deficits in composite score and had significantly more deficits in response time compared to old TIA patients.

adults compared to younger adults [7]. Additional evidence suggests that increased bilateral activity in older adults is seen in posterior regions as well [20,21,32]. In two separate studies, Reuter-Lorenz found that PFC and parietal activity became more bilateral in older adults compared to younger adults for both spatial and verbal working memory tasks [39,40]. To our knowledge the HAROLD model has not been tested directly with lesion data. We hypothesized that acute unilateral stroke to either hemisphere would disrupt the performance of old patients in a verbal working memory task.

### 4.1. *Age and hemispheric effects*

We measured performance in four different components of our working memory task: accuracy, encoding time, response time, and a composite score. In addition, we compared these measurements across all verbal loads, and at the low and high verbal loads alone. If stroke patients displayed deficits in any one of these components we considered the stroke-affected area to be necessary for comparable performance to the TIA patients. Several results from the verbal item-recognition task appear to be consistent with the HAROLD model. We compared the effect of stroke to either hemisphere on task performance to test whether older adults need both hemispheres to perform a verbal working memory task. If the bilateral activation observed in functional neuroimaging studies of working memory represents the recruitment of essential brain areas for performance, then we hypothesized stroke to either hemisphere should result in performance impairment in a verbal working memory task. Our results are consistent with this hypothesis (Figs 2 and 3). Overall, there appears to be bihemispheric deficits on multiple measures in older stroke patients.

Our hypothesis that older stroke patients with damage to either the right or left hemisphere would have deficits in performance was also supported when considered separately (Figs 4 and 5). Across all verbal loads both old right and old left stroke patients had deficits in some measure of performance. This supports the HAROLD model as it suggests that older patients need both hemispheres intact in order to successfully perform the verbal WM task. Interestingly, old left stroke patients did not have deficits in the composite score, but old right patients did. Also, only the old right stroke patients had deficits at the high verbal load. This suggests that older individuals may rely on the right hemisphere more so than on the left hemisphere for verbal working memory, although both hemispheres are needed to perform successfully. Previous studies have shown right PFC recruitment at high WM loads [37,43]. It has been posited that the bilateral activity observed in older adults is due to the recruitment of areas used by younger adults only at high loads and that older adults need to recruit these additional areas at much lower loads than younger adults [9,34]. Overall, these data suggest that older subjects do indeed need both hemispheres, with a high reliance on the right hemisphere, to perform a verbal working memory task.

The increase in bilateral brain activity observed in older adults is thought to represent necessary involvement of additionally recruited areas, a process known as compensation. The idea that the observed bilateral activity is compensatory has been supported by the finding that older subjects that had more bilateral PFC activity performed better than older subjects that displayed less bilateral PFC activity [8]. The hypothesis that bilateral activity represents compensation has further been refined as one of the tenets of the Scaffolding Theory of Aging and Cognition, or STAC model [34]. This model proposes that the observed bilateral activity in older adults represents "the recruitment of additional circuitry that shores up declining structures whose functioning has become noisy, inefficient, or both (p. 183)." The inability to recruit these additional areas, due to neural insults such as stroke, would impair performance in older individuals compared to older individuals that retain the ability to recruit these areas. This could explain the results present here. In addition, the data presented here support the idea that the increase in bilateral activation in older adults is compensatory. In fact, the data presented here suggests that the verbal working memory network may in fact be more localized to the right hemisphere in older adults, as suggested by the fact that older adults with stroke to the right hemisphere in our study were consistently more impaired than older adults with left unilateral stroke.

### 4.2. *Speed-accuracy tradeoff*

An additional interesting finding was that young stroke patients had deficits in accuracy, while old stroke patients had deficits in response time. This pattern emerged when comparing all young stroke patients and all old stroke patients to age-matched controls, as well as in many of our unilateral stroke and age comparisons. One explanation for the different deficits observed in young and old stroke patients is the speed-accuracy trade-off (for review, see [57]). Older subjects have been shown to perform slower, but more accurately than younger subjects in many different tasks [4, 51]. This phenomenon could explain the results seen in the present study even though in this study performance was compared to age-matched controls. In order for older patients to perform accurately it is possible that a further slowing of response time was needed due to the stroke. This would be consistent with the hypothesis put forth by Grady and colleagues that the over-activations observed in older adults could represent the older adults' compensatory attempt to enhance accuracy at the expense of response time [19]. Similarly, if young subjects sacrifice accuracy for response time, those with stroke require additional decrements in accuracy in order to respond quickly. Age-related reduction in processing speed has been proposed to be the main cause of age-related cognitive deficits [48] and most of the age-related variance observed in working memory tasks is associated with the integrity of white matter tracts [10]. If older healthy adults are impaired in response time compared to young adults, then stroke could further impair response time in old stroke patients. For young stroke patients, white matter tracts unaffected by age are reflected by normal response time, therefore the effect of stroke manifests itself in accuracy deficits, although younger stroke patients did have impairments in response time at the high verbal load. Rypma and D'Esposito propose that the neural efficiency hypothesis explains the slower processing speed observed in older subjects [45]. This theory posits that young subjects respond faster than old subjects because they need to recruit fewer areas to accurately complete the task. Aging degrades the integrity of the connections between areas, resulting in older subjects recruiting additional areas to complete the task which slows processing time. Older subjects that do not recruit additional areas perform even slower. Our finding that old stroke patients had more deficits in response time than old TIA patients supports the neural efficiency hypothesis. For old patients, stroke potentially prevents the recruitment of additional brain areas that are available to TIA patients. This leads to further slowing of processing time. For young stroke patients, the neural efficiency model hypothesizes that the recruitment of additional areas slows processing speed. In our study, unilateral stroke to either hemisphere could prevent such recruitment. This could explain why no response time deficits were observed in young stroke patients except at the high verbal load. This suggests that stroke preferentially impairs accuracy at lower levels of cognitive demand compared to response time.

### *4.3. Methodological issues and limitations*

Lesion studies provide the means to test the necessity of brain areas that are shown to be active during specific tasks. While fMRI and other functional imaging techniques allow the correlation of brain activity to task performance, lesion studies allow us to determine what areas of the brain are essential in the performance of a task. Although there have been studies on the effects of PFC and parietal lesions on WM function in humans, no lesion studies have directly tested the hypothesis that older adults need more bilateral involvement than younger adults in tasks primarily associated with one hemisphere [1,14,25,54,55]. In addition, this study is unique from many lesion studies in two important ways. First, acute stroke patients were used as opposed to chronic stroke patients. One concern with lesion studies is the reorganization of normal brain functions that occurs over time in response to brain damage [31]. The use of acute stroke patients circumvents this problem and allows for the evaluation of the effects of the lesion before substantial reorganization can occur. However, the use of acute stroke patients presents different issues. Among these are the stressors that accompany hospitalization and the vascular risk factors associated with stroke. To control for this we enrolled transient ischemic attack patients (TIA) as our control group. The inclusion of TIA patients is the second important distinction of this study. In addition to controlling for hospitalization stressors, the use of TIA patients as controls also takes into account the vascular risk factors and medication histories that are shared between acute stroke and TIA patients. To measure relative performance, we compared performance of old and young acute stoke patients to performance of age-matched TIA patients. Any differences in performances between these groups should be due to the lesion resulting from the stroke, as all other factors were controlled for with the TIA patients.

This study has inherent limitations. First, the use of extreme age groups might improve the ability to detect differences. However, we chose a cut-off of 50 in order to increase participation in the study based on the finding that white matter volume begins declining at this time [18]. Secondly, by characterizing stroke patients by affected-hemisphere we have necessarily grouped heterogeneous stroke sites together. The particular aim of this study was to test the necessity of a hemisphere as a whole, and assumed that strokes to any location in a hemisphere would equally affect working memory performance. Due to limited recruitment of young stroke patients, we were unable to compare performance of patients with stroke to the left or right hemisphere alone. An additional confound is the use of patients with dominant hand weakness. Unfortunately, information regarding how many subjects chose to use their dominant hand is not available. These patients were included to increase participation in this study. Issues such as dominant hand weakness are unavoidable in acute stroke patients. The subject-paced nature of the experiment was due to these issues. It's interesting to note that older stroke patients showed reaction time deficits while younger stroke patients showed deficits in accuracy in comparison to their respective age-matched controls. Accuracy effects should not be a consequence of non-dominant hand use. Likewise, due to the subject paced design of the study, non-dominant hand use should affect both encoding time and response time, irrespective of load. Encoding deficits were only observed at the low loads whereas response time deficits were more prevalent, suggesting the lesions had different effects on the different sub-processes of the task. Given the number of patients in our study, testing for specific stroke location's effect on reaction time is difficult due to power. Future studies should explore the effect of unilateral stroke to more restricted areas (e.g. frontal and/or parietal cortices), and in younger stroke patients in a more controlled manner.

### *4.4. Conclusion*

This study provides evidence for strong bilateral involvement in older patients for verbal WM, a process that is left-lateralized in younger adults [24,35,49]. This validates the Harold model of increased bilateral activity in older adults as being essential and also helps to extend this to areas posterior to the PFC for verbal WM. The results from our verbal WM task support this model and advance the idea that the increased bilateral activity measured in older adults represents essential recruitment of bilateral areas. Although we employed a task that is known to involve fronto-parietal brain regions, we do not predict that these results are specific to this task, as the HAROLD model applies to several cognitive functions [7]. We also present evidence that stroke impairs processing speed in old adults and accuracy in young adults, suggesting that stroke impairs different aspects of performance in old and young adults. In addition to the HAROLD model, these results are consistent with other models of cognitive aging, including the STAC model and the Neural Efficiency model [34,45,46]. Future studies are needed to further validate and potentially consolidate these models of cognitive aging in order for us to better understand age-related reorganization of essential cognitive functions.

## Acknowledgments

Supported by grants UL1RR025011 from the Clinical and Translational Science Award (CTSA) program of the National Center for Research Resources (NCRR), RO1DC05375, NS047691; RC1MH090912 NIMH ARRA Challenge Grant, and T32-GM007507 training grant, National Institutes of Health (NIH).

## References

[1] J.V. Baldo and N.F. Dronkers, The role of inferior parietal and inferior frontal cortex in working memory, *Neuropsychology* **20** (2006), 529–538.
[2] C. Beaulieu, A. de Crespigny, D.C. Tong, M.E. Moseley, G.W. Albers and M.P. Marks, Longitudinal magnetic resonance imaging study of perfusion and diffusion in stroke: evolution of lesion volume and correlation with clinical outcome, *Ann Neurol* **46** (1999), 568–578.
[3] T.J. Bellis, T. Nicol and N. Kraus, Aging affects hemispheric asymmetry in the neural representation of speech sounds, *J Neurosci* **20** (2000), 791–797.
[4] G. Brebion, Working memory, language comprehension, and aging: four experiments to understand the deficit, *Exp Aging Res* **29** (2003), 269–301.
[5] R. Cabeza, C.L. Grady, L. Nyberg, A.R. McIntosh, E. Tulving, S. Kapur, J.M. Jennings, S. Houle and F.I. Craik, Age-related differences in neural activity during memory encoding and retrieval: a positron emission tomography study, *J Neurosci* **17** (1997), 391–400.
[6] R. Cabeza, Cognitive neuroscience of aging: contributions of functional neuroimaging, *Scand J Psychol* **42** (2001), 277–286.
[7] R. Cabeza, Hemispheric asymmetry reduction in older adults: the HAROLD model, *Psychol Aging* **17** (2002), 85–100.
[8] R. Cabeza, N.D. Anderson, J.K. Locantore and A.R. McIntosh, Aging gracefully: compensatory brain activity in high-performing older adults, *Neuroimage* **17** (2002), 1394–1402.
[9] K.A. Cappell, L. Gmeindl and P.A. Reuter-Lorenz, Age differences in prefontal recruitment during verbal working memory maintenance depend on memory load, *Cortex* **46** (2010), 462–473.
[10] R.A. Charlton, T.R. Barrick, I.N. Lawes, H.S. Markus and R.G. Morris, White matter pathways associated with working memory in normal aging. Cortex, 2009.
[11] M. D'Esposito, G.K. Aguirre, E. Zarahn, D. Ballard, R.K. Shin and J. Lease, Functional MRI studies of spatial and nonspatial working memory, *Brain Res Cogn Brain Res* **7** (1998), 1–13.
[12] M. D'Esposito, Functional neuroimaging of cognition, *Semin Neurol* **20** (2000), 487–498.
[13] M. D'Esposito, B.R. Postle and B. Rypma, Prefrontal cortical contributions to working memory: evidence from event-related fMRI studies, *Exp Brain Res* **133** (2000), 3–11.

[14] M. D'Esposito, J.W. Cooney, A. Gazzaley, S.E. Gibbs and B.R. Postle, Is the prefrontal cortex necessary for delay task performance? Evidence from lesion and FMRI data, *J Int Neuropsychol Soc* **12** (2006), 248–260.

[15] S.M. Daselaar, J. Browndyke and R. Cabeza, Functional neuroimaging of cognitive aging. In: Cabeza R, Kingstone A (eds) Handbook of functional neuroimaging and cognition. MIT Press, Cambridge, MA, 2006.

[16] D.L. Dickstein, J.H. Morrison and P.R. Hof, Neuropathology of Aging. In: Jagust W, D'Esposito M (eds) Imaging the Aging Brain, Oxford University Press, New York, New York, 2009.

[17] Y. Ge, R.I. Grossman, J.S. Babb, M.L. Rabin, L.J. Mannon and D.L. Kolson, Age-related total gray matter and white matter changes in normal adult brain. Part I: volumetric MR imaging analysis, *AJNR Am J Neuroradiol* **23** (2002), 1327–1333.

[18] A. Giorgio, L. Santelli, V. Tomassini, R. Bosnell, S. Smith, N. De Stefano and H. Johansen-Berg, Age-related changes in grey and white matter structure throughout adulthood, *Neuroimage* **51** (2010), 943–951.

[19] C.L. Grady, J.M. Maisog, B. Horwitz, L.G. Ungerleider, M.J. Mentis, J.A. Salerno, P. Pietrini, E. Wagner and J.V. Haxby, Age-related changes in cortical blood flow activation during visual processing of faces and location, *J Neurosci* **14** (1994), 1450–1462.

[20] C.L. Grady, A.R. McIntosh, B. Horwitz and S.I. Rapoport, Age-related changes in the neural correlates of degraded and nondegraded face processing, *Cognitive Neuropsychology* **217** (2000), 165–186.

[21] C.L. Grady, L.J. Bernstein, S. Beig and A.L. Siegenthaler, The effects of encoding task on age-related differences in the functional neuroanatomy of face memory, *Psychol Aging* **17** (2002), 7–23.

[22] F.M. Gunning-Dixon, R.C. Gur, A.C. Perkins, L. Schroeder, T. Turner, B.I. Turetsky, R.M. Chan, J.W. Loughead, D.C. Alsop, J. Maldjian and R.E. Gur, Age-related differences in brain activation during emotional face processing, *Neurobiol Aging* **24** (2003), 285–295.

[23] A.E. Hillis, R.J. Wityk, P.B. Barker, N.J. Beauchamp, P. Gailloud, K. Murphy, O. Cooper and E.J. Metter, Subcortical aphasia and neglect in acute stroke: the role of cortical hypoperfusion, *Brain* **125** (2002), 1094–1104.

[24] S. Kapur, R. Rose, P.F. Liddle, R.B. Zipursky, G.M. Brown, D. Stuss, S. Houle and E. Tulving, The role of the left prefrontal cortex in verbal processing: semantic processing or willed action? *Neuroreport* **5** (1994), 2193–2196.

[25] M. Koenigs, A.K. Barbey, B.R. Postle and J. Grafman, Superior parietal cortex is critical for the manipulation of information in working memory, *J Neurosci* **29** (2009), 14980–14986.

[26] Z. Li, A.B. Moore, C. Tyner and X. Hu, Asymmetric connectivity reduction and its relationship to "HAROLD" in aging brain, *Brain Res* **1295** (2009), 149–158.

[27] D.J. Madden, W.L. Whiting, J.M. Provenzale and S.A. Huettel, Age-related changes in neural activity during visual target detection measured by fMRI, *Cereb Cortex* **14** (2004), 143–155.

[28] E.A. Maguire and C.D. Frith, Aging affects the engagement of the hippocampus during autobiographical memory retrieval, *Brain* **126** (2003), 1511–1523.

[29] R. Manenti, M. Cotelli and C. Miniussi, Successful physiological aging and episodic memory: a brain stimulation study, *Behavioural Brain Research* **216** (2011), 153–158.

[30] M.P. Milham, K.I. Erickson, M.T. Banich, A.F. Kramer, A. Webb, T. Wszalek and N.J. Cohen, Attentional control in the aging brain: insights from an fMRI study of the stroop task, *Brain Cogn* **49** (2002), 277–296.

[31] N.G. Muller and R.T. Knight, The functional neuroanatomy of working memory: contributions of human brain lesion studies, *Neuroscience* **139** (2006), 51–58.

[32] K.A. Nielson, S.A. Langenecker and H. Garavan, Differences in the functional neuroanatomy of inhibitory control across the adult life span, *Psychol Aging* **17** (2002), 56–71.

[33] A.M. Owen, C.E. Stern, R.B. Look, I. Tracey, B.R. Rosen and M. Petrides, Functional organization of spatial and nonspatial working memory processing within the human lateral frontal cortex, *Proc Natl Acad Sci U S A* **95** (1998), 7721–7726.

[34] D.C. Park and P.A. Reuter-Lorenz, The adaptive brain: aging and neurocognitive scaffolding, *Annu Rev Psychol* **60** (2009), 173–196.

[35] E. Paulesu, C.D. Frith and R.S. Frackowiak, The neural correlates of the verbal component of working memory, *Nature* **362** (1993), 342–345.

[36] L.E. Philipose, H. Alphs, V. Prabhakaran and A.E. Hillis, Testing conclusions from functional imaging of working memory with data from acute stroke, *Behav Neurol* **18** (2007), 37–43.

[37] V. Prabhakaran, K. Narayanan, Z. Zhao and J.D. Gabrieli, Integration of diverse information in working memory within the frontal lobe, *Nat Neurosci* **3** (2000), 85–90.

[38] V. Prabhakaran, S.P. Raman, M.R. Grunwald, A. Mahadevia, N. Hussain, H. Lu, P.C. Van Zijl and A.E. Hillis, Neural substrates of word generation during stroke recovery: the influence of cortical hypoperfusion, *Behav Neurol* **18** (2007), 45–52.

[39] P.A. Reuter-Lorenz, J. Jonides, E.E. Smith, A. Hartley, A. Miller, C. Marshuetz and R.A. Koeppe, Age differences in the frontal lateralization of verbal and spatial working memory revealed by PET, *J Cogn Neurosci* **12** (2000), 174–187.

[40] P.A. Reuter-Lorenz, S. SE, A. Hartley and R.A. Koeppe, Neurocognitive ageing of storage and executive processes, *European Journal of Cognitive Psychology* **13** (2001), 257–278.

[41] C. Rorden and H.O. Karnath, Using human brain lesions to infer function: a relic from a past era in the fMRI age? *Nat Rev Neurosci* **5** (2004), 813–819.

[42] S. Rossi, C. Miniussi, P. Pasqualetti, C. Babiloni, P.M. Rossini and S.F. Cappa, Age-related functional changes of prefrontal cortex in long-term memory: a repetitive transcranial magnetic stimulation study, *J Neurosci* **24** (2004), 7939–7944.

[43] B. Rypma and M. D'Esposito, The roles of prefrontal brain regions in components of working memory: effects of memory load and individual differences, *Proc Natl Acad Sci U S A* **96** (1999), 6558–6563.

[44] B. Rypma, V. Prabhakaran, J.E. Desmond, G.H. Glover and J.D. Gabrieli, Load-dependent roles of frontal brain regions in the maintenance of working memory, *Neuroimage* **9** (1999), 216–226.

[45] B. Rypma and M. D'Esposito, Isolating the neural mechanisms of age-related changes in human working memory, *Nat Neurosci* **3** (2000), 509–515.

[46] B. Rypma, J.S. Berger, H.M. Genova, D. Rebbechi and M. D'Esposito, Dissociating age-related changes in cognitive strategy and neural efficiency using event-related fMRI, *Cortex* **41** (2005), 582–594.

[47] E. Salmon, M. Van der Linden, F. Collette, G. Delfiore, P. Maquet, C. Degueldre, A. Luxen and G. Franck, Regional brain activity during working memory tasks, *Brain* **119**(Pt 5) (1996), 1617–1625.

[48] T.A. Salthouse, The processing-speed theory of adult age differences in cognition, *Psychol Rev* **103** (1996), 403–428.

[49] E.E. Smith, J. Jonides and R.A. Koeppe, Dissociating verbal and spatial working memory using PET, *Cereb Cortex* **6** (1996), 11–20.

[50] E.E. Smith and J. Jonides, Neuroimaging analyses of human working memory, *Proc Natl Acad Sci U S A* **95** (1998), 12061–12068.

[51] G.A. Smith and N. Brewer, Slowness and age: speed-accuracy mechanisms, *Psychol Aging* **10** (1995), 238–247.

[52] S. Sternberg, High-speed scanning in human memory, *Science* **153** (1966), 652–654.

[53] C.D. Takahashi, L. Der Yeghiaian and S.C. Cramer, Stroke recovery and its imaging, *Neuroimaging Clin N Am* **15** (2005), 681–695, xii.

[54] A. Tsuchida and L.K. Fellows, Lesion evidence that two distinct regions within prefrontal cortex are critical for n-back performance in humans, *J Cogn Neurosci* **21** (2009), 2263–2275.

[55] E. Volle, S. Kinkingnehun, J.B. Pochon, K. Mondon, M. Thiebaut de Schotten, M. Seassau, H. Duffau, Y. Samson, B. Dubois and R. Levy, The functional architecture of the left posterior and lateral prefrontal cortex in humans, *Cereb Cortex* **18** (2008), 2460–2469.

[56] T.D. Wager and E.E. Smith, Neuroimaging studies of working memory: a meta-analysis, *Cogn Affect Behav Neurosci* **3** (2003), 255–274.

[57] W.A. Wickelgren, Speed-Accuracy Tradeoff and Information Processing Dynamics, *Acta Psychologica* **41** (1977), 67–85.
